# Influence of Composition on the Environmental Impact of a Cast Aluminum Alloy

**DOI:** 10.3390/ma9060412

**Published:** 2016-05-25

**Authors:** Patricia Gómez, Daniel Elduque, Judith Sarasa, Carmelo Pina, Carlos Javierre

**Affiliations:** 1 BSH Electrodomésticos España, S.A., Avda. de la Industria, 49, Zaragoza 50016, Spain; patricia.gomez@bshg.com (P.G.); carmelo.pina@bshg.com (C.P.); 2 i+ (i3A), Department of Mechanical Engineering, University of Zaragoza, C/María de Luna, 3, Zaragoza 50018, Spain; carlos.javierre@unizar.es; 3 Department of Chemical Engineering and Environmental Technologies, Environmental Sciences Institute (IUCA), University of Zaragoza, C/María de Luna, 3, Zaragoza 50018, Spain; jsarasa@unizar.es

**Keywords:** life cycle assessment, material composition, environmental impact, aluminum alloy, luminaire housing

## Abstract

The influence of alloy composition on the environmental impact of the production of six aluminum casting alloys (Al Si12Cu1(Fe), Al Si5Mg, Al Si9Cu3Zn3Fe, Al Si10Mg(Fe), Al Si9Cu3(Fe)(Zn) and Al Si9) has been analyzed. In order to perform a more precise environmental impact calculation, Life Cycle Assessment (LCA) with ReCiPe Endpoint methodology has been used, with the EcoInvent v3 AlMg3 aluminum alloy dataset as a reference. This dataset has been updated with the material composition ranges of the mentioned alloys. The balanced, maximum and minimum environmental impact values have been obtained. In general, the overall impact of the studied aluminum alloys varies from 5.98 × 10^−1^ pts to 1.09 pts per kg, depending on the alloy composition. In the analysis of maximum and minimum environmental impact, the alloy that has the highest uncertainty is AlSi9Cu3(Fe)(Zn), with a range of ±9%. The elements that contribute the most to increase its impact are Copper and Tin. The environmental impact of a specific case, an LED luminaire housing made out of an Al Si12Cu1(Fe) cast alloy, has been studied, showing the importance of considering the composition. Significant differences with the standard datasets that are currently available in EcoInvent v3 have been found.

## 1. Introduction

Nowadays there is great social concern about the protection of the environment. Problems like climate change, the greenhouse effect or acid rain are consequences of environmental issues that affect our daily life. In that regard, enterprises have made great efforts to design their products over the last few years to produce goods that preserve environmental resources and reduce the amount of waste and environmental impact.

The ecodesign concept originated with the aim of prevention in the design stage, instead of correction, as most of the environmental impacts are defined when the product idea is conceived and the product is designed [1,2]. At the design stage, material selection takes an important role on the environmental performance, as its affects the whole product life cycle: raw material acquisition, manufacturing process, transportation, and also end of life [3,4].

Many examples of ecodesigned products illustrate this process [5], in areas such as automobiles, bicycles or lighting [6,7], increasing their market competitiveness [8].

Several European policies have been devoted to promoting ecodesign (EuP, 2005/32/CE [9] and ErP, 2009/125/CE [10]) and reducing the use of toxic substances (RoHS, 2002/95/CE [11] and REACH, 1907/2006 [12]). Also, several standards have been created with the aim of introducing the criteria of minimum environmental impact on the development of products (ISO 14006) [13].

An adequate methodology to analyze the environmental impacts is Life Cycle Assessment (LCA), which evaluates the environmental impact of a material, product, process or service, identifying the main types of environmental impact throughout the life cycle [14,15]. LCA has been applied to a wide range of different products and materials such as commercial biodegradable polymers [16], concrete [17,18], luminaires [19], lubricants [20] or cars based on aluminum, steel, magnesium and plastic [21].

This article is focused on evaluating the environmental impact of the production of aluminum alloys, currently used in a wide range of applications [22]. Aluminum has high strength, high electrical and thermal conductivity, low density, good ductility and high hardness [23]. Although there are many techniques and manufacturing processes for aluminum such as extrusion, rolling, stamping or bending, this study is focused on aluminum casting [24,25].

Many authors and experts have used the LCA to analyze the environmental impact of several aluminum products such as beverage cans [26], the use of aluminum in the automotive sector [27], or its use in buildings [28]. Furthermore, in the last few decades aluminum alloys have been widely used in the industry, due to their enhanced properties such as a lower melting point, strength or hardness [29,30]. A great variety of properties can be obtained depending on the elements added to the alloy. For example, silicon improves the fluidity of the alloy and at the same time reduces its melting temperature, while tin increases the machinability of the alloys [29]. However, the alloying elements should be chosen carefully. Although these elements generally improve the properties of the material, they also have an influence on the environmental impact of the alloy.

LCA can be used as a potential tool to help businesses or governments to assess their supply chains and to analyze the environmental impact of materials or components. It also provides information about the presence of critical raw materials, thanks to the development of a detailed Life Cycle Inventory [31,32]. A critical material is defined for several reasons: shortage or supply risk, economic vulnerability or ecological risk [33,34,35,36,37]. For all these reasons, the composition of an alloy must be studied while taking into consideration the presence of critical raw materials and how they can affect the total environmental impact. This research came up when LCA was applied to a real case of an LED luminaire housing made of an aluminum casting alloy. The compositions of aluminum alloys are defined by standards that include elements intentionally added to improve properties, and also those impurities that cannot be removed during alloy production. There are a wide range of LCA studies which use datasets from databases such as EcoInvent to evaluate the environmental impact of metals alloys like aluminum, steel, copper or titanium [38,39,40]. However, it was found that the composition of aluminum casting alloys did not correspond to the ones available in the EcoInvent database. This fact could influence the calculated environmental impact.

Therefore, the main objective of this work is to assess the environmental impact of several aluminum alloys and evaluate the influence that their composition has on the environment. To this end, the differences between several alloys have been analyzed using EcoInvent v3 data [24]. A real case study of an LED luminaire housing is presented to show those differences.

## 2. Materials and Methods

### 2.1. Composition and Properties of Aluminum Casting Alloys

This work is based on the EcoInvent methodology, using EcoInvent v3 datasets customized with different alloying elements, in order to achieve a more precise environmental impact assessment calculation. In this work, six different alloys that are commonly used for casting have been selected (Table 1). The composition of aluminum casting alloys is defined by standards, such as the European EN 1706:2011 [41], the Japanese H5302 [42], the British BS 1490 [43], the French NF EN 755-3-2008 [44], or the American AA ASTM B179-14 [45,46]. The corresponding equivalences of the selected alloys under different standards are shown in Table 1. The differences in the alloy compositions influence their properties, which are shown in Table 2.

### 2.2. Dataset Improvement Methodology for Aluminum Casting Alloys

The production processes of each aluminum alloy have been analyzed, taking into account the composition of each studied alloy. The methodology used by EcoInvent for the dataset “Aluminium alloy, AlMg3 {RER}| production” has been applied in this research. This dataset, which can be used as a proxy for this type of aluminum casting alloy, has a 3% content of magnesium and small amounts of other metals [24]. These data allows an analysis of the total material consumption. The overall amount of raw materials needed for the production processes corresponds to the materials that appear in the final alloy and also the overall material consumption considering auxiliary raw material consumption. The compositions ranges of all the selected alloys are shown in Section 3.

The EcoInvent v3 AlMg3 dataset has been updated with the material composition of the mentioned alloys. As the composition of an alloy is defined by ranges, a balanced composition has been calculated in order to obtain a defined representative composition of that alloy. This balanced composition calculation method consists of the following phases:

- Firstly, the alloying elements that have a range are identified for each alloy.
- Secondly, for each alloy and for each of these alloying elements defined with a range, the average value between the minimum and the maximum values of each range is calculated. This leads to a balanced composition that is representative of an alloy whose alloying elements percentages are in the middle of the allowed range.
- Finally, the average percentage values for each alloying element of the composition are considered. For the analyzed alloys, it has been checked that the sum of the alloying element percentages in each alloy adds up 100%.

All the information of the alloys is processed while taking into account EcoInvent system characterization introducing it into EcoInvent v3 dataset and updating it.

In this way, the LCA of aluminum casting alloys was performed and compared with EcoInvent’s original dataset, in order to evaluate the influence of the composition on the environmental impact.

### 2.3. LCA Methodology

#### 2.3.1. Goal and Scope Definition

The aim of this LCA is to quantify the impact of several aluminum casting alloys, analyzing the influence of the composition on the environmental impact. ISO 14040 standards of LCA have been applied [47].

#### 2.3.2. Functional Unit

The definition of a functional unit is essential in life cycle assessment. In this paper, the functional unit has been defined as the production of 1 kg of aluminum alloy, taking into consideration the composition of the alloying, energy consumption and the end of life of the waste produced in the production processes.

#### 2.3.3. System Boundaries

The scope of this research is to focus on the study of the environmental impact of the production of an aluminum cast alloy and to investigate the influence of the variation of the composition.

**Figure 1:**
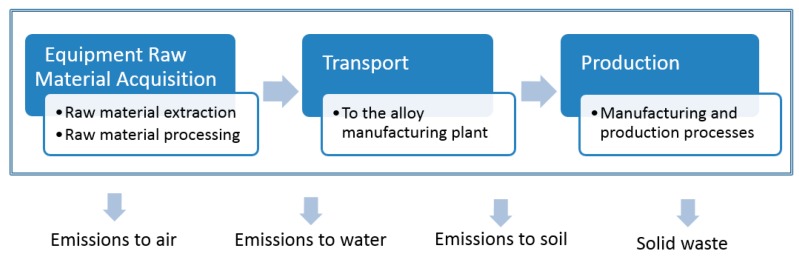
System boundaries.

The LCA has been developed to evaluate the environmental impact of each aluminum alloy considering the composition of alloying elements and also impurities, focusing on the stages shown in Figure 1. Following EcoInvent’s methodology, a material loss of 1.48% during production is assumed. Following these methodology, market (GLO) datasets have been used to consider transportation of the raw material from the average providers to the alloy manufacturing plant.

#### 2.3.4. Inventory Data and Assumptions

The life cycle inventory has been developed by means of EcoInvent v3, one of the most important databases implemented by the Swiss Centre for Life Cycle Inventories [48], using the attributional modeling approach “Allocation, ecoinvent default”, where market datasets are considered a mix between primary and secondary sources.

The LCA has been modeled with SimaPro 8.0.3.14, developed by Pré Consultants [49]. Furthermore, the LCA was developed with ReCiPe methodology. This methodology is useful for designers because of its ease of interpretation. ReCiPe methodology was developed as an endpoint attempt to align the CML 2002 midpoint and the Eco-indicator 99 systems [50].

## 3. Life Cycle Inventory

In order to compare each alloy with EcoInvent’s dataset, the life cycle inventories of all the selected aluminum alloys have been developed. Table 3 shows a detailed inventory of the composition of each aluminum alloy, including the one that corresponds to EcoInvent’s v3 dataset. The composition of the studied alloys is given with respect to 1 kg of aluminum alloy. It can be seen that the quantity of some elements varies considerably from some alloys to others. This is the case with nickel, titanium, lead or tin. These materials are not considered in EcoInvent’s AlMg3 aluminum alloy dataset; however, they are used in all of the other studied alloys. Chromium is only included in EcoInvent and alloy #5 (Al Si9Cu3(Fe)(Zn)) datasets, but none of the other alloys have this element in their composition.

On the other hand, the main material, aluminum, and some elements like silicon, copper and magnesium are present in a range of quantities for each alloy. The ranges in the composition will be considered to obtain not only a balanced value of the environmental impact, as explained in Section 2, but also the minimum and the maximum environmental impact of the range of each alloy. Table 4 shows the balanced composition present in 1 kg of each aluminum alloy.

Table 5 shows the studied aluminum alloys and the material inputs needed to manufacture 1 kg of each alloy. These data are used to calculate the amount of aluminum alloy raw input used, considering not only 1 kg of input but also all the raw materials inputs to obtain the functional unit. An input of raw material acquisition of 1.0148 kg for each kilogram of manufactured aluminum alloy, as established by EcoInvent’s methodology, has been considered to develop the LCA, as explained in Section 2.3.3.

The most relevant EcoInvent datasets that have been used to characterize the inputs of the aluminum alloys are shown in Table 6. All of them have been selected following EcoInvent’s guidelines [24].

## 4. Results

Once the life cycle inventories were introduced in SimaPro, the balanced, minimum and maximum environmental impacts were obtained for the alloys. These results have been analyzed in order to study the influence of the material composition. Finally, the results of environmental impact of a LED luminaire aluminum housing are shown to demonstrate the composition influence with a real example.

### 4.1. Analysis of the Balanced Environmental Impact of the Aluminum Alloys

Table 7 shows the environmental impact calculations of each alloy in ReCiPe points by kilogram, considering the balanced value of the materials that are given as a range in Table 3. In general, it can be said that the highest environmental impact is produced by alloy #3 (Al Si9Cu3Zn3Fe) with 1.01 pts, followed by alloy #5 (Al Si9Cu3(Fe)(Zn)) with 9.44 × 10^−1^ pts. The minimum environmental impact is produced by alloy #6 (Al Si9) with an environmental impact of 6.09 × 10^−1^ pts; Furthermore, it can be observed that the alloying elements that create the highest environmental impact are copper, silicon and tin, elements commonly used in aluminum alloys [29]. The alloys which have the highest environmental impact are those with the highest copper and tin percentage values. In contrast, the EcoInvent original dataset produces only 6.36 × 10^−1^ points of environmental impact, a low value, because EcoInvent’s composition does not include tin and its quantity of silicon is the lowest of the studied alloys.

As mentioned before, alloy #3 (Al Si9Cu3Zn3Fe) creates the highest environmental impact in points by kilogram, where Copper and Tin composition percentages contribute in a significant way to this impact, with values of 2.02 × 10^−1^ and 1.84 × 10^−1^ points per kilogram of alloy, respectively. Also in this alloy, aluminum represents 80.95% of the composition and its environmental impact corresponds to 27.74% of the total environmental impact of the alloy. This alloy contains 3% copper, which has a significant effect on the strength and hardness of the alloy and generates 20.06% of the total environmental impact of the alloy. Similarly, although tin in the alloy is only 0.25% of the composition, it accounts for 18.21% of the environmental impact of the alloy. Tin is used to reduce friction when it is a requirement in some applications [29].

Chromium, magnesium and silicon, which are used in several alloys, show a high environmental impact. EU studies carried out in 2013 include these elements in the EU critical material list, regarding economic vulnerability, shortage and ecological risk. [33,51]. They therefore have a high importance to the EU, and a high risk associated with their supply risk and economic importance. On the other hand, lead and iron contribute to reducing impact values, owing to their low environmental impact values in the studied alloys. Both materials contribute positively to the characteristics of the alloys. Lead improves the machinability of the alloy and iron increases the mechanical strength [52,53]. 

All the studied alloys present an uncertainty owing to the range values in some components. The maximum and the minimum possible environmental impacts of all the analyzed aluminum alloys are calculated in Subsection 4.2 and Subsection 4.3.

### 4.2. Analysis of the Maximum Environmental Impact of the Aluminum Alloys

Table 8 shows the maximum environmental impact produced by the studied aluminum alloys in points by kilogram. This maximum impact is calculated taking into account all the possible combinations of the compositions ranges for each alloy (Table 3). The compositions that create this maximum impact are shown as Supplementary Materials (Table S1).

When the maximum environmental impact of each alloy is compared with the balanced value, it can be seen that, in general, it increases between 1% and 8.9%. The highest difference is observed for alloy #5 (Al Si9Cu3(Fe)(Zn)), as there is an increment of 8.35 × 10^−2^ pts (8.9%), from 9.44 × 10^−1^ to 1.03 pts. Copper and silicon are the components with the highest absolute increase. The maximum environmental impact due to copper has increased 6.74 × 10^−2^ pts from the balanced value, which was 2.02 × 10^−1^ pts. The contribution of environmental impact caused by silicon is lower but 1.62 × 10^−2^ pts higher than the balanced value.

Alloy #3 (Al Si9Cu3Zn3Fe) also shows an increment of environmental impact of 8.5% from balanced to maximum values. This impact is mainly caused by copper, which has increased 6.74 × 10^−2^ pts from the balanced composition value. By contrast, the alloy that shows the lowest increase is alloy #1(Al Si12Cu1(Fe)) with only a 1% increase.

### 4.3. Analysis of the Minimum Environmental Impact of the Aluminum Alloys

The minimum environmental impact produced by the studied aluminum alloys in points by kilogram is shown in Table 9. As in the previous subsection, this minimum impact is calculated by combinations of the composition ranges for each alloy (Table 3). The compositions that create this minimum impact are shown as Supplementary Materials (Table S2).

As in the study of the maximum environmental impact, alloy #5 (Al Si9Cu3(Fe)(Zn)) contributes to the highest percentage decrease of environmental impact from balanced to minimum values, with a decrease of 8.9%, from 9.44 × 10^−1^ to 8.60 × 10^−1^ pts. This impact is mainly decreased by magnesium, which decreases 9.49 × 10^−3^ pts from the balanced composition value. By contrast, the alloys that show the lowest decrease are alloy #2 (Al Si5Mg) and alloy #4 (Al Si10Mg(Fe)), whereas in alloy #5 the environmental impact is mainly decreased by magnesium in both alloy #2 and #4.

Finally, once the balanced, maximum and minimum environmental impacts of all aluminum alloys were analyzed, it can be seen that those alloys with the highest environmental impact (alloy #3 and alloy #5) are the ones that show the highest difference between maximum and minimum environmental impact, with values of 1.72 × 10^−1^ and 1.67 × 10^−1^ pts, respectively.

### 4.4. Study of the Environmental Impact of an LED Luminaire Housing

**Figure 2:**
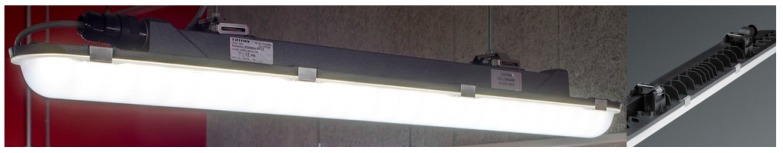
LED luminaire.

One of the applications of these alloys is to manufacture LED luminaire housings. LED luminaires generate a significant amount of heat that has to be dissipated away from the electronic components to ensure proper performance and avoid failure. That is achieved in weatherproof luminaires using a 1.4 kg aluminum housing. This housing is manufactured out of an aluminum alloy into a mold using a die casting machine with a locking force of 12,000 kN. Of all the studied alloys, the luminaire under study is made out of alloy #1 (Al Si12Cu1(Fe)) which offers good manufacturability and machinability for the component. It has an excellent fluidity that makes it suitable for thin-wall casting (Figure 2), thanks to a high silicon content. Other relevant alloying elements are nickel, which is used to increase the strength, and tin, which increases machinability. Focusing on the alloy properties, and taking into account the use for an LED luminaire housing, the selected alloy offers the lowest expansion coefficient and the highest elastic limit of all the six analyzed alloys. It also provides an adequate range of thermal conductivity, which is useful to enhance the thermal behaviour of the LED system.

Due to its specific composition, the environmental impact of this component, should not be calculated with standard datasets such as “Aluminium, cast alloy {GLO}” or “Aluminium alloy, AlMg3 {RER}”, available in EcoInvent, as it has been demonstrated that the differences in environmental impact are relevant. Table 10 compares the impact with EcoInvent’s proxy and the selected alloy. There is an increase of between 0.158 and 0.179 pts per housing if compared with the AlMg3 alloy; moreover, the environmental impact is up to 127% higher than the standard aluminum cast alloy described by EcoInvent. This is a clear example of the influence of the alloy composition on the environmental impact results.

In order to show the magnitude of the environmental impact of this case study, the Global Warming Potential (GWP) expressed in kg CO_2_ eq., is also given in the tables of this subsection. Under this impact category, the calculated carbon footprint decreases between 3.28 and 3.57 kg CO_2_ eq. per housing when compared with the AlMg3 alloy. For GWP, the impact of the analyzed alloy decreases, as some of the alloying elements, such as copper or tin, have a significant impact on ReCiPe endpoint methodology, and a relatively lower one in this category.

To show broader analysis of this case study, the system boundaries for this case study have been expanded to include the manufacturing process in of this LED luminaire housing in a die casting machine. Table 11 shows the same information as Table 10, but considering the environmental impact of the produced part (materials plus production processes).

From these results it can be said that on the one hand, as previously shown in Table 10, using balanced alloy #1 allowed us to obtain a more precise environmental impact, which in this case is lower in GWP than the value obtained with the AlMg3 EcoInvent dataset, but higher under ReCiPe endpoint methodology. On the other hand, analyzing the environmental impact considering the production process shows the great relevance of material production, and therefore its composition, on the overall environmental impact. Focusing on alloy #1, the material production generates between 83.8% and 84.1% of the total impact under the ReCiPe methodology, and between 73% and 73.9% under Global Warming Potential.

## 5. Conclusions

This article highlights the importance of considering the material composition in order to properly assess the environmental impact of aluminum casting alloys, allowing engineers not only to compare these alloys based on cost or mechanical properties, but also to determine a more precise environmental impact. The influence of the alloy composition has been analyzed by means of Life Cycle Assessment, using the EcoInvent AlMg3 aluminum alloy dataset as a reference.

As standards define aluminum casting alloys with composition ranges, once the EcoInvent v3 dataset was updated with the material composition of the mentioned alloys, the balanced, maximum and minimum environmental impact values have been obtained using these ranges.

In general, the overall impact of the studied balanced aluminum alloys (Al Si12Cu1(Fe), Al Si5Mg, Al Si9Cu3Zn3Fe, Al Si10Mg(Fe), Al Si9Cu3(Fe)(Zn) and Al Si9), using the ReCiPe endpoint methodology, varies from 6.09 × 10^−1^ pts to 1.01 pts per kg, depending on the alloy composition. These results are significantly different from the EcoInvent AlMg3 dataset, in which the impact is 6.36 × 10^−1^ pts per kg. Some of the alloying elements that contribute to increased environmental impact are copper and tin. Furthermore, most of the studied alloys have raw materials such as magnesium, chromium and silicon, which are considered critical raw materials by the European Union due to their supply risk and economic importance.

When analyzing the maximum and minimum environmental impact of the aluminum alloys, the impact varies from 5.98 × 10^−1^ pts to 1.09 pts per kg. The alloy that has the highest uncertainty is alloy #5 (Al Si9Cu3(Fe)(Zn)), with a range of ±9%, due to copper and silicon.

The LED luminaire Al Si12Cu1(Fe) housing case study has shown the relevance of taking the composition into account, with average differences from EcoInvent’s proxy of 0.17 pts in ReCiPe and 3.4 kg CO_2_ eq. per housing in GWP. Also, the importance of the material impact on the production process has been shown.

## Figures and Tables

**Table 1:** Designation and nomenclature of the studied alloys.

Alloy	EN AC 1760 Symbolic	EN AC 1706 Numeric	JIS H5302	BS 1490	NF 755-3	ASTM B179	UNS
#1	Al Si12Cu1(Fe)	47,100	ADC1	LM 20	A-S12U	–	–
#2	Al Si5Mg	–	–	LM 8	A-SAG-Y	–	–
#3	Al Si9Cu3Zn3Fe	–	ADC10	LM 24	AS9U3	380	A90380
#4	Al Si10Mg(Fe)	43,400	ADC3	–	–	–	–
#5	Al Si9Cu3(Fe)(Zn)	46,500	ADC10Z	LM 24	A-S9U3X	–	–
#6	Al Si9	44,400	–	–	AS-9	–	–

**Table 2:** Main properties of the studied alloys.

Mechanical Characteristics	Alloy #1	Alloy #2	Alloy #3	Alloy #4	Alloy #5	Alloy #6
Expansion coefficient (10^−6^/k)	20.0	23.0	21.5	21.0	21.0	21.0
Thermal conductivity (w/mK)	120–150	146–180	109	130–150	110–120	130–150
Electrical conductivity (MS/m)	15–20	4.5–5.3	7.5	16–21	13–17	16–22
Tensile strength, Rm (MPa)	240	140	170	240	240	220
Elastic limit, Rp0.2% (MPa)	140	100	100	140	140	120
Elongation (%)	1	1	1	1	<1	2
Brinell hardness (HBW)	70	60	75	70	80	55

**Table 3:** Material composition for 1 kg of aluminum alloy.

Material (kg)	EcoInvent	Alloy #1		Alloy #2		Alloy #3		Alloy #4		Alloy #5		Alloy #6	
Silicon	4.00 × 10^−3^	1.10 × 10^−1^	1.35 × 10^−1^	4.00 × 10^−2^	5.50 × 10^−2^	8.00 × 10^−2^	1.10 × 10^−1^	9.00 × 10^−2^	1.10 × 10^−1^	8.00 × 10^−2^	1.10 × 10^−1^	8.00 × 10^−2^	1.10 × 10^−1^
Iron	4.00 × 10^−3^	1.30 × 10^−2^		6.00 × 10^−3^		1.30 × 10^−2^		1.00 × 10^−2^		6.00 × 10^−3^	1.20 × 10^−2^	5.50 × 10^−3^	
Copper	1.01 × 10^−3^	1.00 × 10^−2^		1.00 × 10^−3^		2.00 × 10^−2^	4.00 × 10^−2^	1.00 × 10^−3^		2.00 × 10^−2^	4.00 × 10^−2^	8.00 × 10^−4^	
Manganese	5.01 × 10^−3^	5.00 × 10^−3^		6.00 × 10^−3^		5.50 × 10^−3^		5.50 × 10^−3^		5.50 × 10^−3^		5.00 × 10^−3^	
Magnesium	3.01 × 10^−2^	2.00 × 10^−3^		5.00 × 10^−3^	8.00 × 10^−3^	5.00 × 10^−4^	5.50 × 10^−3^	2.00 × 10^−3^	5.00 × 10^−3^	1.50 × 10^−3^	5.50 × 10^−3^	1.00 × 10^−3^	
Nickel	0.00	5.00 × 10^−3^		1.00 × 10^−3^		5.50 × 10^−3^		1.50 × 10^−3^		5.50 × 10^−3^		5.00 × 10^−4^	
Zinc	2.00 × 10^−3^	5.00 × 10^−3^		1.00 × 10^−3^		3.00 × 10^−2^		1.50 × 10^−3^		3.00 × 10^−2^		1.50 × 10^−3^	
Titanium	0.00	2.00 × 10^−3^		2.00 × 10^−3^		2.50 × 10^−3^		2.00 × 10^−3^		2.00 × 10^−3^		1.50 × 10^−3^	
Lead	0.00	1.50 × 10^−3^		1.00 × 10^−3^		3.50 × 10^−3^		1.50 × 10^−3^		3.50 × 10^−3^		5.00 × 10^−4^	
Tin	0.00	1.00 × 10^−3^		5.00 × 10^−4^		2.50 × 10^−3^		5.00 × 10^−4^		1.50 × 10^−3^		5.00 × 10^−4^	
Chromium	3.01 × 10^−3^	0.00		0.00		0.00		0.00		1.50 × 10^−3^		0.00	
Aluminum	9.51 × 10^−1^	8.21 × 10^−1^	8.46 × 10^−1^	9.19 × 10^−1^	9.37 × 10^−1^	7.82 × 10^−1^	8.37 × 10^−1^	8.85 × 10^−1^	8.62 × 10^−1^	7.83 × 10^−1^	8.43 × 10^−1^	8.73 × 10^−1^	9.03 × 10^−1^
Total	1.00	1.00		1.00		1.00		1.00		1.00		1.00	

**Table 4:** Balanced material composition for 1 kg of aluminum alloy.

Material (kg)	EcoInvent	Alloy #1	Alloy #2	Alloy #3	Alloy #4	Alloy #5	Alloy #6
Silicon	0.0040	0.1225	0.0475	0.0950	0.1000	0.0950	0.0950
Iron	0.0040	0.0130	0.0060	0.0130	0.0100	0.0090	0.0055
Copper	0.0010	0.0100	0.0010	0.0300	0.0010	0.0300	0.0008
Manganese	0.0050	0.0050	0.0060	0.0055	0.0055	0.0055	0.0050
Magnesium	0.0301	0.0020	0.0065	0.0030	0.0035	0.0035	0.0010
Nickel	0.0000	0.0050	0.0010	0.0055	0.0015	0.0055	0.0005
Zinc	0.0020	0.0050	0.0010	0.0300	0.0015	0.0300	0.0015
Titanium	0.0000	0.0020	0.0020	0.0025	0.0020	0.0020	0.0015
Lead	0.0000	0.0015	0.0010	0.0035	0.0015	0.0035	0.0005
Tin	0.0000	0.0010	0.0005	0.0025	0.0005	0.0015	0.0005
Chromium	0.0030	0.0000	0.0000	0.0000	0.0000	0.0015	0.0000
Aluminum	0.9509	0.8330	0.9275	0.8095	0.8730	0.8130	0.8882
Total	1.0000	1.0000	1.0000	1.0000	1.0000	1.0000	1.0000

**Table 5:** Balanced material composition inputs for 1 kg of aluminum alloy.

Material (kg)	EcoInvent	Alloy #1	Alloy #2	Alloy #3	Alloy #4	Alloy #5	Alloy #6
Silicon	0.0041	0.1243	0.0482	0.0964	0.1015	0.0964	0.0964
Iron	0.0041	0.0132	0.0061	0.0132	0.0101	0.0091	0.0056
Copper	0.0010	0.0101	0.0010	0.0304	0.0010	0.0304	0.0008
Manganese	0.0051	0.0051	0.0061	0.0056	0.0056	0.0056	0.0051
Magnesium	0.0305	0.0020	0.0066	0.0030	0.0036	0.0036	0.0010
Nickel	0.0000	0.0051	0.0010	0.0056	0.0015	0.0056	0.0005
Zinc	0.0020	0.0051	0.0010	0.0304	0.0015	0.0304	0.0015
Titanium	0.0000	0.0020	0.0020	0.0025	0.0020	0.0020	0.0015
Lead	0.0000	0.0015	0.0010	0.0036	0.0015	0.0036	0.0005
Tin	0.0000	0.0010	0.0005	0.0025	0.0005	0.0015	0.0005
Chromium	0.0031	0.0000	0.0000	0.0000	0.0000	0.0015	0.0000
Aluminum	0.9650	0.8453	0.9412	0.8215	0.8859	0.8250	0.9013
Total	1.0148	1.0148	1.0148	1.0148	1.0148	1.0148	1.0148

**Table 6:** EcoInvent dataset selection.

Material	Dataset
Copper	Copper {GLO}| market for | Alloc Def, U
Magnesium	Magnesium {GLO}| market for | Alloc Def, U
Aluminum	Aluminium, cast alloy {GLO}| market for | Alloc Def, U
Silicon	Silicon, metallurgical grade {GLO}| market for | Alloc Def, U
Manganese	Manganese {GLO}| market for | Alloc Def, U
Zinc	Zinc {GLO}| market for | Alloc Def, U
Chromium	Chromium {GLO}| market for | Alloc Def, U
Nickel	Nickel, 99.5% {GLO}| market for | Alloc Def, U
Lead	Lead {GLO}| market for | Alloc Def, U
Tin	Tin {GLO}| market for | Alloc Def, U

**Table 7:** Environmental impact of 1 kg of the studied aluminum alloys.

Material	EcoInvent	Alloy #1	Alloy #2	Alloy #3	Alloy #4	Alloy #5	Alloy #6
Total (pts)	6.36 × 10^−1^	7.62 × 10^−1^	6.12 × 10^−1^	1.01	6.36 × 10^−1^	9.44 × 10^−1^	6.09 × 10^−1^
Silicon	4.26 × 10^−3^	1.32 × 10^−1^	5.13 × 10^−2^	1.03 × 10^−1^	1.08 × 10^−1^	1.03 × 10^−1^	1.03 × 10^−1^
Iron	1.03 × 10^−3^	3.40 × 10^−3^	1.57 × 10^−3^	3.40 × 10^−3^	2.61 × 10^−3^	2.35 × 10^−3^	1.44 × 10^−3^
Copper	6.68 × 10^−3^	6.74 × 10^−2^	6.74 × 10^−3^	2.02 × 10^−1^	6.74 × 10^−3^	2.02 × 10^−1^	5.39 × 10^−3^
Manganese	4.37 × 10^−2^	4.43 × 10^−2^	5.32 × 10^−2^	4.88 × 10^−2^	4.88 × 10^−2^	4.88 × 10^−2^	4.43 × 10^−2^
Magnesium	1.40 × 10^−1^	9.49 × 10^−3^	3.08 × 10^−2^	1.42 × 10^−2^	1.66 × 10^−2^	1.66 × 10^−2^	4.74 × 10^−3^
Nickel	0.00	3.66 × 10^−2^	7.32 × 10^−3^	4.02 × 10^−2^	1.10 × 10^−2^	4.02 × 10^−2^	3.66 × 10^−3^
Zinc	2.01 × 10^−3^	5.10 × 10^−3^	1.02 × 10^−3^	3.06 × 10^−2^	1.53 × 10^−3^	3.06 × 10^−2^	1.53 × 10^−3^
Titanium	0.00	1.26 × 10^−3^	1.26 × 10^−3^	1.58 × 10^−3^	1.26 × 10^−3^	1.26 × 10^−3^	9.47 × 10^−4^
Lead	0.00	9.34 × 10^−5^	6.23 × 10^−5^	2.18 × 10^−4^	9.34 × 10^−5^	2.18 × 10^−4^	3.11 × 10^−5^
Tin	0.00	7.34 × 10^−2^	3.67 × 10^−2^	1.84 × 10^−1^	3.67 × 10^−2^	1.10 × 10^−1^	3.67 × 10^−2^
Chromium	1.30 × 10^−2^	0.00	0.00	0.00	0.00	6.60 × 10^−3^	0.00
Aluminum	3.24 × 10^−1^	2.88 × 10^−1^	3.21 × 10^−1^	2.80 × 10^−1^	3.02 × 10^−1^	2.81 × 10^−1^	3.07 × 10^−1^

**Table 8:** Maximum environmental impact of 1 kg of the studied aluminum alloys.

Material	Alloy #1	Alloy #2	Alloy #3	Alloy #4	Alloy #5	Alloy #6
Total (pts)	7.72 × 10^−1^	6.24 × 10^−1^	1.09	6.50 × 10^−1^	1.03	6.21 × 10^−1^
Silicon	1.46 × 10^−1^	5.94 × 10^−2^	1.19 × 10^−1^	1.19 × 10^−1^	1.19 × 10^−1^	1.19 × 10^−1^
Iron	3.40 × 10^−3^	1.57 × 10^−3^	3.40 × 10^−3^	2.61 × 10^−3^	3.14 × 10^−3^	1.44 × 10^−3^
Copper	6.74 × 10^−2^	6.74 × 10^−3^	2.70 × 10^−1^	6.74 × 10^−3^	2.70 × 10^−1^	5.39 × 10^−3^
Manganese	4.43 × 10^−2^	5.32 × 10^−2^	4.88 × 10^−2^	4.88 × 10^−2^	4.88 × 10^−2^	4.43 × 10^−2^
Magnesium	9.49 × 10^−3^	3.79 × 10^−2^	2.61 × 10^−2^	2.37 × 10^−2^	2.61 × 10^−2^	4.74 × 10^−3^
Nickel	3.66 × 10^−2^	7.32 × 10^−3^	4.02 × 10^−2^	1.10 × 10^−2^	4.02 × 10^−2^	3.66 × 10^−3^
Zinc	5.10 × 10^−3^	1.02 × 10^−3^	3.06 × 10^−2^	1.53 × 10^−3^	3.06 × 10^−2^	1.53 × 10^−3^
Titanium	1.26 × 10^−3^	1.26 × 10^−3^	1.58 × 10^−3^	1.26 × 10^−3^	1.26 × 10^−3^	9.47 × 10^−4^
Lead	9.34 × 10^−5^	6.23 × 10^−5^	2.18 × 10^−4^	9.34 × 10^−5^	2.18 × 10^−4^	3.11 × 10^−5^
Tin	7.34 × 10^−2^	3.67 × 10^−2^	1.84 × 10^−1^	3.67 × 10^−2^	1.10 × 10^−1^	3.67 × 10^−2^
Chromium	0.00	0.00	0.00	0.00	6.60 × 10^−3^	0.00
Aluminum	2.84 × 10^−1^	3.17 × 10^−1^	2.70 × 10^−1^	2.98 × 10^−1^	2.71 × 10^−1^	3.02 × 10^−1^

**Table 9:** Minimum environmental impact of 1 kg of the studied aluminum alloys.

Material (pts)	Alloy #1	Alloy #2	Alloy #3	Alloy #4	Alloy #5	Alloy #6
Total (pts)	7.53 × 10^−1^	6.00 × 10^−1^	9.22 × 10^−1^	6.22 × 10^−1^	8.60 × 10^−1^	5.98 × 10^−1^
Silicon	1.19 × 10^−1^	4.32 × 10^−2^	8.64 × 10^−2^	9.72 × 10^−2^	8.64 × 10^−2^	8.64 × 10^−2^
Iron	3.40 × 10^−3^	1.57 × 10^−3^	3.40 × 10^−3^	2.61 × 10^−3^	1.57 × 10^−3^	1.44 × 10^−3^
Copper	6.74 × 10^−2^	6.74 × 10^−3^	1.35 × 10^−1^	6.74 × 10^−3^	1.35 × 10^−1^	5.39 × 10^−3^
Manganese	4.43 × 10^−2^	5.32 × 10^−2^	4.88 × 10^−2^	4.88 × 10^−2^	4.88 × 10^−2^	4.43 × 10^−2^
Magnesium	9.49 × 10^−3^	2.37 × 10^−2^	2.37 × 10^−3^	9.49 × 10^−3^	7.11 × 10^−3^	4.74 × 10^−3^
Nickel	3.66 × 10^−2^	7.32 × 10^−3^	4.02 × 10^−2^	1.10 × 10^−2^	4.02 × 10^−2^	3.66 × 10^−3^
Zinc	5.10 × 10^−3^	1.02 × 10^−3^	3.06 × 10^−2^	1.53 × 10^−3^	3.06 × 10^−2^	1.53 × 10^−3^
Titanium	1.26 × 10^−3^	1.26 × 10^−3^	1.58 × 10^−3^	1.26 × 10^−3^	1.26 × 10^−3^	9.47 × 10^−4^
Lead	9.34 × 10^−5^	6.23 × 10^−5^	2.18 × 10^−4^	9.34 × 10^−5^	2.18 × 10^−4^	3.11 × 10^−5^
Tin	7.34 × 10^−2^	3.67 × 10^−2^	1.84 × 10^−1^	3.67 × 10^−2^	1.10 × 10^−1^	3.67 × 10^−2^
Chromium	0.00	0.00	0.00	0.00	6.60 × 10^−3^	0.00
Aluminum	2.92 × 10^−1^	3.24 × 10^−1^	2.89 × 10^−1^	3.06 × 10^−1^	2.91 × 10^−1^	3.12 × 10^−1^

**Table 10:** Environmental impact of the material of a LED luminaire housing.

Material Dataset	LED Luminaire Housing Material ReCiPe Impact (pts)	LED Luminaire Housing Material CO_2_ IPCC 2013 GWP (100 y) (kg CO_2_ eq.)
Aluminium, cast alloy {GLO}	4.778 × 10^−1^	4.472
Aluminium alloy, AlMg3 {RER}	9.037 × 10^−1^	9.748
Minimum impact alloy #1 Al Si12Cu1(Fe)	1.056	6.182
Balanced alloy #1 Al Si12Cu1(Fe)	1.070	6.328
Maximum impact alloy #1 Al Si12Cu1(Fe)	1.083	6.473

**Table 11:** Environmental impact of the material plus manufacturing process of an LED luminaire housing.

Material Dataset	Produced LED Luminaire Housing (Material + Manufacturing) ReCiPe Impact (pts)	Produced LED Luminaire Housing (Material + Manufacturing) CO_2_ IPCC 2013 GWP (100 y) (kg CO_2_ eq.)
Aluminium, cast alloy {GLO}	6.826 × 10^−1^	6.759
Aluminium alloy, AlMg3 {RER}	1.109	1.204 × 10
Minimum impact alloy #1 Al Si12Cu1(Fe)	1.261	8.469
Balanced alloy #1 Al Si12Cu1(Fe)	1.275	8.615
Maximum impact alloy #1 Al Si12Cu1(Fe)	1.288	8.760

## Supplementary Materials

The following are available online at www.mdpi.com/1996-1944/9/6/412/s1.
