# Supplementary material for: Influence of Composition on the Environmental Impact of a Cast Aluminum Alloy
Source: Materials (Basel). 2016 May 25;9(6):412. doi: 10.3390/ma9060412 (PMC5456811; doi:10.3390/ma9060412)
Supplement: Supplementary file 1 [file materials-09-00412-s001.pdf]

Supplementary Materials: Influence of Composition on the Environmental Impact of a Cast Aluminum Alloy

Patricia Gómez, Daniel Elduque, Judith Sarasa, Carmelo Pina and Carlos Javierre

Table S1. Material composition of 1 kg of the studied aluminum alloys for maximum environmental impact.

Material (kg)	Alloy #1	Alloy #2	Alloy #3	Alloy #4	Alloy #5	Alloy #6
Total (kg)	1.00	1.00	1.00	1.00	1.00	1.00
Silicon	1.35×10^{-1}	5.50×10^{-2}	1.10×10^{-1}	1.10×10^{-1}	1.10×10^{-1}	1.10×10^{-1}
Iron	1.30×10^{-2}	6.00×10^{-3}	1.30×10^{-2}	1.00×10^{-2}	1.20×10^{-2}	5.50×10^{-3}
Copper	1.00×10^{-2}	1.00×10^{-3}	4.00×10^{-2}	1.00×10^{-3}	4.00×10^{-2}	8.00×10^{-4}
Manganese	5.00×10^{-3}	6.00×10^{-3}	5.50×10^{-3}	5.50×10^{-3}	5.50×10^{-3}	5.00×10^{-3}
Magnesium	2.00×10^{-3}	8.00×10^{-3}	5.50×10^{-3}	5.00×10^{-3}	5.50×10^{-3}	1.00×10^{-3}
Nickel	5.00×10^{-3}	1.00×10^{-3}	5.50×10^{-3}	1.50×10^{-3}	5.50×10^{-3}	5.00×10^{-4}
Zinc	5.00×10^{-3}	1.00×10^{-3}	3.00×10^{-2}	1.50×10^{-3}	3.00×10^{-2}	1.50×10^{-3}
Titanium	2.00×10^{-3}	2.00×10^{-3}	2.50×10^{-3}	2.00×10^{-3}	2.00×10^{-3}	1.50×10^{-3}
Lead	1.50×10^{-3}	1.00×10^{-3}	3.50×10^{-3}	1.50×10^{-3}	3.50×10^{-3}	5.00×10^{-4}
Tin	1.00×10^{-3}	5.00×10^{-4}	2.50×10^{-3}	5.00×10^{-4}	1.50×10^{-3}	5.00×10^{-4}
Chromium	0.00	0.00	0.00	0.00	1.50×10^{-3}	0.00
Aluminum	8.21×10^{-1}	9.19×10^{-1}	78.20×10^{-1}	8.62×10^{-1}	7.83×10^{-1}	8.73×10^{-1}
Maximum environmental impact per 1 kg (pts)	7.72×10^{-1}	6.24×10^{-1}	1.09	6.50×10^{-1}	1.03	6.21×10^{-1}

Table S2. Material composition of 1 kg of the studied aluminum alloys for minimum environmental impact.

Material (kg)	Alloy #1	Alloy #2	Alloy #3	Alloy #4	Alloy #5	Alloy #6
Total (kg)	1.00	1.00	1.00	1.00	1.00	1.00
Silicon	1.10×10^{-1}	4.00×10^{-2}	8.00	9.00×10^{-2}	8.00×10^{-2}	8.00×10^{-2}
Iron	1.30×10^{-2}	6.00×10^{-3}	1.30	1.00×10^{-2}	6.00×10^{-3}	5.50×10^{-3}
Copper	1.00×10^{-2}	1.00×10^{-3}	2.00	1.00×10^{-3}	2.00×10^{-2}	8.00×10^{-4}
Manganese	5.00×10^{-3}	6.00×10^{-3}	5.50×10^{-1}	5.50×10^{-3}	5.50×10^{-3}	5.00×10^{-3}
Magnesium	2.00×10^{-3}	5.00×10^{-3}	5.00×10^{-2}	2.00×10^{-3}	1.50×10^{-3}	1.00×10^{-3}
Nickel	5.00×10^{-3}	1.00×10^{-3}	5.50×10^{-1}	1.50×10^{-3}	5.50×10^{-3}	5.00×10^{-4}
Zinc	5.00×10^{-3}	1.00×10^{-3}	3.00	1.50×10^{-3}	3.00×10^{-2}	1.50×10^{-3}
Titanium	2.00×10^{-3}	2.00×10^{-3}	2.50×10^{-1}	2.00×10^{-3}	2.00×10^{-3}	1.50×10^{-3}
Lead	1.50×10^{-3}	1.00×10^{-3}	3.50×10^{-1}	1.50×10^{-3}	3.50×10^{-3}	5.00×10^{-4}
Tin	1.00×10^{-3}	5.00×10^{-4}	2.50×10^{-1}	5.00×10^{-4}	1.50×10^{-3}	5.00×10^{-4}
Chromium	0.00	0.00	0.00	0.00	1.50×10^{-3}	0.00
Aluminum	8.46×10^{-1}	9.37×10^{-1}	8.37×10	8.85×10^{-1}	8.43×10^{-1}	9.03×10^{-1}
Minimum environmental impact per 1 kg (pts)	7.53×10^{-1}	6.00×10^{-1}	9.22×10^{-1}	6.22×10^{-1}	8.60×10^{-1}	5.98×10^{-1}
